# Should basic health economics principles be taught during medical school in the UK?

**DOI:** 10.3402/meo.v20.29541

**Published:** 2015-10-12

**Authors:** Muhammad A. Ashraf, Yusuf Sherwani, Muhammad Najim, Maroof Ahmed, Riham Rabee, Osama Al-Jibury, Faisal Al-Mayahi, Aaniya Ahmed

**Affiliations:** Department of Medicine, Imperial College London, London, UK

The landscape of the NHS is undergoing significant change in recent times. A £30 billion funding gap is expected to be opened up by 2020/2021 (1), intensifying fears of the economic crisis surrounding the institution. This brings forth the undeniable fact that money, or resource, management has become critical to achieve sustainability within the NHS.

As medical students who recently completed a BSc in Management, we found that the health economics module in particular allowed us to gain invaluable skills beyond the stethoscope, granting us an insight as to complexity and difficult nature of financial decisions in any healthcare system. Most doctors coming out of medical school will be completely oblivious to this other side of medicine; how, then, can they be expected to rise to the challenges ahead?

Failing to prepare is preparing to fail. The current medical school curricula in the UK are guilty of neglecting this increasingly vital aspect of healthcare in the NHS. We strongly believe that an increased effort must be made to integrate health economics into the curricula in the same manner that ethics has become a core element of the course; indeed, the foundation of resource allocation is entwined with key ethical principles of distributive justice (2).

Possible approaches to achieve this could be through a series of online seminars. These might be supplemented by a series of workshops in which students can engage in more practical application of the knowledge gained. Such models have already been proposed (2), but have not been expanded on and rolled out on a national scale.

Interestingly, the concept of fully student-run clinics has been part of the medical education system in the States for over a decade, with over 100 such clinics in the country now (3). Furthermore, Australia has also recently followed lead with the implementation of its own “REACH” project (4). The benefits of such proposals are two-fold. Alongside the advantage of reducing the burden on other health services, students also gain hands-on experience in practicing not only clinical skills but also more uniquely experience daily management challenges. It allows economic principles such as financing and resource allocation to actually be put into practice.

Whilst the best method to deliver teaching on health economics is unclear and should be explored further, the current need for this education at the undergraduate level is crystal clear. We believe that the success of our future NHS hinges on it.

*Muhammad A. Ashraf* Department of Medicine Imperial College London London, UK Email: muhammad.ashraf10@imperial.ac.uk *Yusuf Sherwani* Department of Medicine Imperial College London London, UK *Muhammad Najim* Department of Medicine Imperial College London London, UK *Maroof Ahmed* Department of Medicine Imperial College London London, UK *Riham Rabee* Department of Medicine Imperial College London London, UK *Osama Al-Jibury* Department of Medicine Imperial College London London, UK *Faisal Al-Mayahi* Department of Medicine Imperial College London London, UK *Aaniya Ahmed* Department of Medicine Imperial College London London, UK
